# Evaluating governance in a clinical and translational research organization

**DOI:** 10.1017/cts.2024.25

**Published:** 2024-02-13

**Authors:** Ingrid Philibert, Amanda Fletcher, Katrina M. Poppert Cordts, Matthew Rizzo

**Affiliations:** Great Plains IDeA CTR, University of Nebraska Medical Center, Omaha, NE, USA

**Keywords:** Clinical and translational research, governance, meta-evaluation, quality improvement

## Abstract

Institutional Development Awards for Clinical and Translational Research (IDeA-CTR) networks, funded by NIH/NIGMS, aim to advance CTR infrastructure to address historically unmet state and regional health needs. Success depends on the response to actionable feedback to IDeA-CTR leadership from network partners and governance groups through annual surveys, interviews, and governance body recommendations. The Great Plains IDeA-CTR applied internal formative meta-evaluation to evaluate dispositions of 172 governance recommendations from 2017 to 2021. Results provided insights to improve the classification and quality of recommendations, credibility of evaluation processes, responsiveness to recommendations, and communications and governance in a complex CTR network comprising multiple coalitions.

## Background

Institutional Development Award (IDeA) Clinical and Translational Research (CTR) network awards from the National Institute of General Medical Sciences (NIGMS), aim to advance institutional infrastructure and personnel to address critical unmet clinical and public health needs across historically underserved states and regions [1]. Meeting this aim depends on systems thinking [2], informed by successive cycles of strategic self-assessment, providing actional feedback for IDeA-CTR formation and growth across a network of regional partners and headquarters, with the aid of governance groups, sound principles of tracking and evaluation (T&E), providing input for effective administration and management of IDeA-CTR network goals, projects, and tasks.

The Great Plains (GP) IDeA-CTR network was established in 2016, with headquarters at the University of Nebraska Medical Center. CTR partners included (1) institutions across Nebraska and the Dakotas that receive technology and research resources and share equipment and expertise with the network; (2) investigators who enhance their CTR skills through scholar and pilot grants, mentoring, and other core services (e.g., biostatistics, informatics); and (3) patients and communities standing to benefit from CTR focus on regional health priorities. Governance is aided by an external advisory committee of national CTR experts, an internal advisory committee of research leaders at participating institutions, a community advisory board made up of members of academia and community organizations, and a Steering Committee including CTR core directors and partner institution representatives.

Effective tracking and evaluation of NIH-funded programs is key to enhancing capacity [3]. We explored the application of internal formative meta-evaluation (IFME) to track and evaluate GP IDeA-CTR disposition of recommendations from the initial funding period (2017–2021), in efforts to improve our IDeA-CTR evaluation process, as outlined below.

## Methods

Meeting IDeA-CTR/NIGMS goals depends on a cycle of feedback, operationalized by a T&E Core, through annual assessments of partners, investigators, patients and communities, and governance groups specified above, and providing critical feedback to the IDeA-CTR’s Principal Investigator (PI), administrative core, and other cores. The T&E core developed and disseminated five annual governance reports between 2017 and 2021. Each report comprised quantitative data on governance effectiveness using a modified internal coalition evaluation (ICE) survey [4,5], narrative comments on the survey, governance interviews, and classification of disposition of recommendations. The modified ICE tool has 18 questions spanning Social Vision, Efficient Practices, Knowledge and Training, Relationships, Participation, and Activities [4,5] (see supplemental information for details). The ICE instrument is among the tools highlighted by the National Academy of Medicine’s report on Assessing Meaningful Community Engagement[6] and by the National Institute of Health’s HEAL Initiative [7].

Meta-evaluation appraises the evaluation process or the resulting outcomes to enhance evaluation quality and credibility [8–10]. This includes summative meta-evaluations by external reviewers that focus on outcomes. In contrast, formative meta-evaluations are conducted by internal evaluators and focus on the evaluation process [10,11]. We deployed Stufflebeam’s internal formative meta-evaluation (IFME) approach for its focus on member engagement, and timely communication and reporting [8], to assess: (1) strengths and opportunities for improvement in responses to governance recommendations; and (2) longitudinal evidence of coalition-building.

T&E staff collaborated with all GP IDeA CTR cores to update information on CTR responses to 172 total recommendations and classify these as (1) enacted/completed as recommended; (2) enacted by alternate approach; (3) scheduled for action upon CTR renewal; and (4) infeasible.

## Results

**Figure 1.**
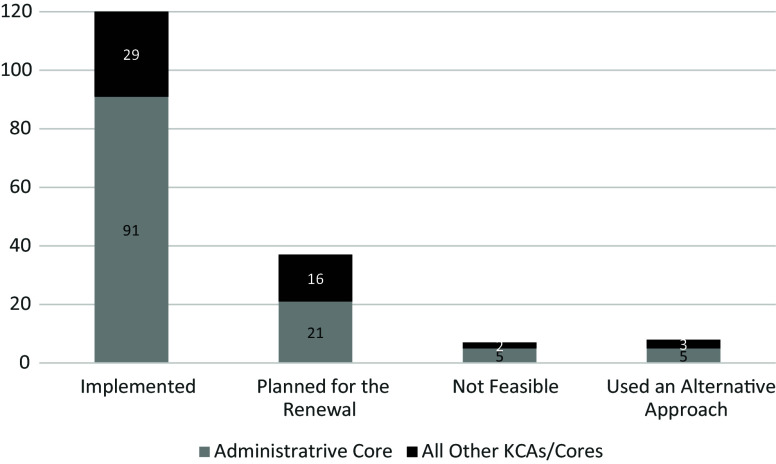
Disposition of all governance recommendations 2017–2021.

Figure 1 shows the disposition of all 172 recommendations after IFME. Of these,120 (69.8%) were enacted; 8 (4.7%) were implemented through alternative approaches that met the recommendation’s intent; 37 (21.5%) were scheduled for action upon grant renewal; and 7 (4.1%) were deemed infeasible due to resource demands, implementation challenges, or limited anticipated benefit (e.g., a data repository for CTR-funded projects was judged infeasible because data varied widely across projects), with low potential for leveraging future research

### Strengths and opportunities

**Figure 2.**
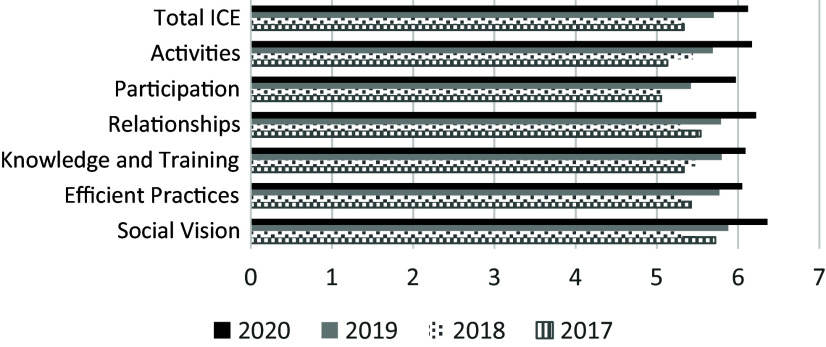
ICE scores 2017–2020 – steering committee, funded faculty and staff.

Results highlighted CTR strengths in response to governance input during network evolution, notwithstanding external challenges, such as the global SARS-CoV-2 (COVID-19) pandemic. In total, 91.3% of the recommendations (157 of 172) were implemented or scheduled for implementation in the renewal, reflecting CTR responsiveness to governance recommendations, and actionable quality of recommendations received. Responsiveness also is reflected in successive improvements in satisfaction with governance reported on all dimensions of the longitudinal data from the ICE survey [3,4] for 2016–2017 and 2020–2021 (Fig. 2).

Use of IFME (through the ICE tool and governance interviews) allowed the CTR to address a small drop in scores for social vision, efficient practices, and relationships between 2017 and 2018. They were attributed to slower- than-anticipated network development after the initial year and rebounded in 2019, with continued growth in subsequent years.

Use of IFME improved the display of information in communications with governance advisory bodies. Tabular data in the 2020–2021 governance report efficiently presented the disposition of responses to all recommendations in one place, closing the feedback loop on all recommendations from the initial funding period. IFME also enabled refinements in the evaluation process, including splitting recommendations into multiple components for analysis and action and making explicit situations where implementation met governance recommendation intent through alternative approaches. An example of implementation using an alternative approach is the response to the recommendation to have unfunded pilot applicants meet with their reviewers. The alternative approach gives applicants the option to discuss their application with BERD consultants or with relevant experts through research studios. Implementation of a recommendation through an alternative approach is reported in the governance report, giving governance groups the opportunity to ask questions and receive clarifying information.

### Evaluation of coalition building

IFME helped us examine how the number and character of annual recommendations related to the six ICE constructs for measuring effective coalitions (Social Vision, Efficient Practices, Knowledge and Training, Relationships, Participation, and Activities; Table 1). Earlier years were characterized by a larger number of recommendations to help the PI and the administrative core establish governance and management infrastructure, and education and training programs, with a focus on the ICE domains of Knowledge, Training, and Relationships. From 2019 to 2021, recommendations were fewer, more complex, and focused on actions in the domains of Participation and Activities, reflecting a maturing network. Recommendations for Participation included the active engagement of partner institutions and support of investigators from different member sites. Recommendations for Activities included the use of de-identified EHR data and the development of a precision medicine program. Actions in the Activities domain also showed a growing focus on the needs of historically and currently underserved populations in the region, including tribal members and rural residents. GP IdeA-CTR initiatives to benefit these groups included expansion of the practice-based research network (PBRN) to 89 sites that help translate research into care to improve the health of communities. An enacted governance recommendation created a vetting process for PBRN study proposals that tasks the PBRN’s board of directors with ensuring that research conducted across the network benefits the GP IdeA-CTR, the PBRN, and local communities.

**Table 1.** Categorizing governance using the ICE attributes of effective coalition

	Social vision		Efficient practices		Knowledge and training		Relationships		Participation		Activities		All		
2019–2021	7	12.5%	8	14.3%	7	12.5%	14	25.0%	7	12.5%	13	23.2%	56	100%	
2017–2018	7	6.1%	18	15.7%	39	33.9%	40	34.8%	6	5.2%	6	5.2%	115	100%	
All years	14	8.1%	26	15.1%	46	26.7%	54	31.4%	13	7.6%	19	11.0%	172	100%	

As shown in Table 1, the ICE construct Efficient Operations was a theme through all five years, including repositioning network assets and resources to address changing needs and priorities, such as (2019–2020) joining the National COVID Cohort Collaborative (N3C) [12].

Analysis of score differences for the CTR’s advisory boards and its steering committee, with the latter having significant representation from network members, showed advisory board scores were slightly lower than those for the steering committee, although differences were not statically significant. Trend analysis showed improvement in scores for both groups since the initial survey in 2017, with Shared Social Vision showing the highest score for both groups, and Efficient Practices and Participation showing the largest gains for both groups.

## Discussion

Demonstrating responsiveness to feedback in an IDeA-CTR requires effective communication that closes network feedback loops [13] with partners, investigators, patients, communities, and governance groups. Doing this can enhance the relevance and credibility of feedback by reducing “noise,” and spacing feedback to allow recommendations to be considered before additional input is provided [14].

*Classifying* recommendations as “implemented using an alternative approach” in meta-evaluation helps improve classification and terminology for reporting, and clarifies reports on implementation responsiveness, communication of results, and comparisons across IDeA-CTRs, through a shared taxonomy of evaluation [15].

Our approach to how the GP IDeA-CTR responded to governance recommendations distinguished between recommendations to improve administrative and governance functions; CTR processes, capabilities, and capacity; and governance evaluation. Meta-evaluation in another CTR used “evaluation utility metrics” (EUMs) to classify whether recommendations were fully or partially adopted, and if they led to actionable changes in program processes or products [16].” Reflecting on the level of influence of a recommendation is germane to all IDeA CTRs. Generalization is challenging as findings depend on the system, culture, and context wherein governance recommendations were generated [16].

IFME offers new ideas for program evaluation across a complex CTR network. Our use of IFME identified efficiency and timeliness of communications with governance groups as opportunities, in line with NIGMS (PAR-20-175: IDeA Program Infrastructure for Clinical and Translational Research) (IDeA-CTR) (U54 - Clinical Trial Optional) (nih.gov). This tasked IDeA-CTRs with assessing short- and long-term aims, including implementation of specific program activities (process), and plans for documenting accomplishments for each budget period and the total award period (outcomes) [1]. A limitation of IFME is that it focuses more on process than outcomes of evaluations. Future refinements can include summative meta-evaluation, focusing more on the influence of recommendations and their ultimate impact on course corrections and success.

## Conclusion

We used IFME to analyze five years of governance recommendations and the CTR’s response and described its evolution in a maturing network. Our findings may help other IDeA programs, funders, and the public gain insights into the value of meta-evaluation in appraising governance in programs serving multiple CTR teams and partners.

## Supporting information

Philibert et al. supplementary material Philibert et al. supplementary material

## Supplementary material

The supplementary material for this article can be found at https://doi.org/10.1017/cts.2024.25.
