# Supplementary material for: Evaluating governance in a clinical and translational research organization
Source: J Clin Transl Sci. 2024 Feb 13;8(1):e42. doi: 10.1017/cts.2024.25 (PMC10928697; doi:10.1017/cts.2024.25)
Supplement: Philibert et al. supplementary material [file S2059866124000256sup001.docx]

***The Internal Coalition Evaluation Instrument Adapted for CTR Use***

**Background:** The Internal Coalition Evaluation (ICE) instrument was developed by Mary Cramer, RN, PhD. It is based on prior work on published attributes of effective coalitions, including the Internal Coalition Outcome Hierarchy (ICOH) model, which focuses on group-process theory based on the assumption that coalitions are microcosms of society that require effective organizational structures to support consensus for a shared vision and achievement of goals.

The original citation is Cramer ME, Atwood JR, Stoner JA. Measuring Community Coalition Effectiveness Using the ICE^©^ Instrument. Public Health Nursing. 2006;23:74-87.

The ICE instrument was adapted for the GP IDeA CTR activities by Dr. Cramer in her role as inaugural director of the tracking and evaluation for the Great Plains IDeA CTR and has been used in the evaluation of governance effectiveness for the four years of the government report open prevents 2017 to 2020/2021.

The adaptations for CTR use consist of focusing solely on leader effectiveness in coalition-building (the original version of the ICE survey included questions for leader and member effectiveness), reducing the number of questions from 28 to 18, and minor adjustments to terminology, such as changing “coalition” to “network.”

**The item stem for all questions is:** GP-CTR leadership and governance function to ensure....

**The answer choices are:** 1 = Strongly Disagree to 7 = Strongly Agree, with 7 denoting optimal performance.

***ICE Domains and Items***

**Shared Social Vision**

1. a shared vision among Network members

2. consensus about the Network mission and purpose

**Efficient Practices**

3. involvement of institutional partners in the work of the Network

4. repositioning of Network assets, competencies, and resources to address the changing needs and priorities

5. resource sharing for translational research investigators across the Network of institutional partners

**Knowledge and Training**

6. provision of education that keeps Network members current on topics and best practices related to clinical and translational research

7. provision of resources to develop new translational research leaders across the Network

8. provision of resources that develop clinical and translational research knowledge among community members

**Relationships**

9. development of positive relations with communities (e.g., Community Advisory Board) in order to engage them in clinical and translational research

10. transparent and respectful relationships among Network members

11. open and transparent communications between Network members

**Participation**

12. involvement of Network members in quality improvement processes, including establishment of priorities and evaluation of goal achievement

13. active participation by Network members in decision-making

14. a sense of inclusivity that respects diverse perspectives and input

15. engagement and support of diverse Network investigators from our institutional partners

**Activities**

16. organizational oversight of the Network including ensuring that goals, activities, and metrics are achieved on a timely basis

17. conflict management when problems about activity implementation arise within the Network

18. provision of clinical and translational research resources that reflect the needs and priorities of Network investigators, including pilots and scholars
